# Physical activity and sedentary behavior in relation to mortality among renal cell cancer survivors

**DOI:** 10.1371/journal.pone.0198995

**Published:** 2018-06-12

**Authors:** Daniela Schmid, Charles E. Matthews, Michael F. Leitzmann

**Affiliations:** 1 Department of Epidemiology and Preventive Medicine, University of Regensburg, Regensburg, Germany; 2 Division of Cancer Epidemiology and Genetics, National Cancer Institute, National Institutes of Health, U.S. Department of Health and Human Services, Bethesda, Maryland, United States of America; University of Zurich, SWITZERLAND

## Abstract

**Background:**

The relations of physical activity and sedentary behavior to mortality risk among patients with renal cell cancer have not yet been evaluated.

**Methods:**

We conducted a prospective cohort study among 667 renal cell cancer survivors aged 50–71 years of the National Institutes of Health (NIH)-AARP Diet and Health Study with a median follow-up time of 7.1 years. Post-diagnosis physical activity, TV viewing, and total sitting time were assessed using self-administered questionnaires. Hazard ratios (HRs) and 95% confidence intervals (CIs) for mortality were estimated using Cox proportional hazards models.

**Results:**

Increasing levels of moderate to vigorous physical activity were related to decreased risk of overall mortality [multivariable-adjusted HRs for <1 hr/wk (reference), 1 to 3 hrs/wk, ≥3 to <7 hrs/wk, and ≥7 hrs/wk = 1.0, 1.16, 0.94, and 0.60 (95% CI = 0.38–0.96; p-trend = 0.03)]. In contrast, television viewing was associated with increased risk of overall mortality in the age- and sex-adjusted model (HR for >4 hrs/d vs. 0 to 2 hrs/d = 1.52, 95% CI = 1.02–2.26; p-trend = 0.04), but the relation was attenuated following further control for other covariates (multivariable-adjusted HR = 1.44, 95% CI = 0.92–2.24; p-trend = 0.11). Total sitting time was unrelated to all-cause mortality.

**Conclusion:**

Among renal cancer patients, moderate to vigorous physical activity is associated with decreased risk of overall mortality. Clinicians should consider discussing the potential benefits of physical activity for longevity among survivors of renal cell cancer.

## Introduction

In 2017, an estimated 63,990 new cases of kidney and renal pelvis cancer occurred in U.S. adults [1]. Although renal cell cancer is known to be the most lethal urologic malignancy [1], the estimated five-year survival rate of kidney and renal pelvis cancer among U.S. adults notably improved from 50% between 1975 and 1977 to 74% between 2004 and 2010, likely a consequence of improved cancer diagnosis and treatment. Therefore, the impact of recognizing modifiable risk factors after diagnosis resulting in improved survival is enormously high.

Obesity, smoking, hypertension, and type 2 diabetes mellitus are considered risk factors for renal cell cancer [2]. A recent meta-analysis [3] and a pooled study of 1.44 million adults [4] showed that physical activity protects against renal cell cancer incidence. Possible biologic mechanisms to explain this link include improved immune function [5] and decreases in chronic inflammation [5], insulin resistance [5], hypertension [6], and lipid peroxidation [7]. Prolonged time spent sitting has been found to be unrelated to renal cell cancer incidence [8], although evidence exists for a positive association between prolonged sitting time and obesity [9–11].

The study of physical activity and sedentary behavior in relation to cancer survival is currently underrepresented though of great clinical significance. Recent data suggest that greater physical activity is associated with lower risks of overall and cause-specific mortality among survivors of breast [12], colon [12], and prostate cancers [13–15]. The sparse literature on television (TV) viewing/leisure sitting time and survival of colorectal [16–18], breast [19], and endometrium cancers [20] accrued to date has, for the most part, not been consistent across studies. The relations of physical activity and sedentary behavior to renal cell cancer survival have not yet been evaluated. Therefore, we conducted a prospective examination of physical activity, TV viewing, and total sitting time after diagnosis to all-cause and cause-specific mortality among individuals diagnosed with renal cell cancer in the large National Institutes of Health (NIH)-AARP Diet and Health Study cohort.

## Methods

### Study population

The NIH—AARP Diet and Health Study established in 1995/1996 is a prospective cohort study that included 566,398 adults aged 50–71 years at the start of the study, residing in six U.S. states or two U.S. metropolitan areas [21]. Covariate information was assessed in the baseline questionnaire in 1995/1996 and risk factor questionnaire, which was sent within six months to those without colon, breast or prostate cancer at baseline (S1 Fig). Exposure information was taken from a follow-up questionnaire filled out by study participants in 2004/2005. Eligible individuals for the present study were cohort members diagnosed with a first primary renal cell cancer who were free of cancer (other than non-melanoma skin cancer) prior to study entry in 1995/1996 and who were diagnosed with renal cell cancer before the follow-up entry (n = 682). We excluded participants with body mass index (BMI)<18.5 kg/m^2^ (n = 10) and those with missing information on exposure from the respective analysis (physical activity analysis: n = 5, TV viewing analysis: n = 45, total sitting analysis: n = 30). Exclusions were also made for those reporting out of range values for overall sitting (≥ 22.5 hrs/d, n = 9).

A total of 667 renal cell cancer survivors remained for the physical activity analysis. For the TV viewing and total sitting analyses, 627 and 633 renal cell cancer survivors were included, respectively.

This study was approved by the Special Studies Institutional Review Board of the U.S. National Cancer Institute and completion of the questionnaires was considered to imply informed consent.

### Assessment of physical activity and sedentary behavior

In the follow-up questionnaire, participants were asked to report the weekly time spent doing moderate to vigorous physical activity over the prior 12 months, choosing from eight exercise and recreational activities such as walking, jogging, tennis, swimming, bicycling, or weight training. Participants chose from 10 duration categories ranging from none to more than 10 h/week for each activity. We collapsed the categories for physical activity into four categories: <1 hr/wk, 1 to <3 hrs/wk, ≥3 to <7 hrs/wk, and ≥7 hrs/wk. This questionnaire also queried about TV or video viewing, sitting or driving in a car, bus, or train, and other sitting (reading, knitting, using a computer). For TV viewing, we created the following categories: 0 to 2 hrs/d, >2 to 4 hrs/d, and >4 hrs/d. Total sitting time was summed across domain-specific questions and divided into three categories: 0 to 5 hrs/d, >5 to 8 hrs/d, and >8 hrs/d.

The physical activity assessment of the follow-up questionnaire shows a reasonable correlation with physical activity energy expenditure as assessed by doubly labeled water (r = 0.33) and the sitting time item is positively correlated with a corresponding assessment based on the activPAL (r = 0.16) [22].

In the risk factor questionnaire, a list of examples of leisure time moderate to vigorous physical activity was provided and study members were asked to report how often they participated in leisure time moderate to vigorous physical activity in the past 10 years. This questionnaire also inquired about hours watching TV/videos or sitting per day over the past 12 months.

### Ascertainment of renal cell cancer incidence and mortality

Histologically confirmed renal cell cancer cases diagnosed between 1995/1996 and 2004/2005 were included. Renal cell cancer cases were identified through probabilistic linkages to eleven state cancer registry databases that has been described in detail previously [23]. Briefly, multiple identifiers were used to improve the probability of true matches in the cancer registries [23]. To determine the accuracy of cancer case ascertainment through cancer registries, a pilot study was conducted in a subsample of the NIH-AARP Diet and Health Study population [23]. The study estimated that the sensitivity of the registries included in the NIH-AARP cohort for case ascertainment is about 90%, with a 4-year lag period between the end of follow-up of cancer incidence and the linkage date [23]. We defined renal cell cancer using the International Classification of Diseases for Oncology, 3rd Edition (ICD-O-3) [24].

Vital status was determined by linkage to the Social Security Administration Death Master File [25] and the National Death Index Plus through December 31, 2011 [26]. End points were mortality from any cause and mortality from renal cell cancer, defined by the ICD-9 and ICD-10 codes.

### Statistical analysis

Cox proportional hazards models were fit to estimate hazard ratios (HRs) and 95% confidence intervals (CIs) for mortality across increasing categories of physical activity, TV viewing, or total sitting. Participants were followed from the date of the follow-up questionnaire entry to either death or December 31, 2011, whichever came first.

We assessed mortality risk in three models: 1) adjusting for age at exposure assessment (continuous), age at diagnosis (continuous), and sex; 2) additionally adjusting for ethnicity (non-Hispanic White, non-Hispanic Black, other, unknown), education (less than 12 years, 12 years, vocational training or some college education, college graduate/postgraduate, unknown), history of diabetes (yes, no), history of hypertension (yes, no), smoking (never smoker, stopped smoking 10 or more years ago, stopped smoking 5–9 years ago, stopped smoking 1–4 years ago, stopped smoking within last year, currently smoking, unknown), alcohol consumption (0, 0.1 to 14.9, ≥15g/d), surgery (yes, no, unknown/missing), chemotherapy (yes, no, unknown/missing), radiation (yes, no, unknown/missing), tumor stage (in situ, localized, regional metastases, distant metastases, unknown/not abstracted/missing), and mutually adjusting for total sitting time and physical activity; and 3) additionally adjusting for BMI (18.5-<25.0 kg/m^2^, 25.0-<30.0 kg/m^2^, 30.0-<35.kg/m^2^, 35-<65 kg/m^2^, missing). All covariates were taken from the baseline questionnaire or risk factor questionnaire (hypertension variable), except BMI and smoking, which were queried about in the follow-up questionnaire. Information on cancer stage and first course of treatment reported within one year of diagnosis was provided by cancer registries.

Because of concerns about possible reverse causation, we performed sensitivity analyses excluding those who died within one year of completing the follow-up questionnaire. We further excluded participants who answered the questionnaire within a year of diagnosis.

Linear trends across increasing categories of physical activity and sedentary behavior were calculated using the median of each category as a continuous variable. We tested for the proportional hazards assumption using Schoenfeld residuals [27]. The P values were two-sided and were considered statistically significant at the 0.05 level. Analyses were performed using SAS version 9.4 (SAS Institute, Cary, NC).

## Results

During a median follow-up time of 7.1 years, we documented 175 deaths for the physical activity analysis and 163 deaths for the TV viewing analysis, of which 57 and 53 were due to renal cell cancer, respectively. Age-standardized characteristics for moderate to vigorous physical activity and TV viewing after diagnosis are shown in Table 1. Participants with greater physical activity levels were less likely to be current smokers and to have a history of diabetes mellitus than the least active participants. By comparison, individuals reporting prolonged TV viewing were more likely to be current smokers and to have histories of diabetes mellitus and hypertension than less sedentary individuals.

**Table 1:** Baseline characteristics of study participants diagnosed with incident renal cell cancer according to increasing levels of post-diagnosis moderate to vigorous physical activity and TV viewing.

Characteristics	Post-diagnosis physical activity				Post-diagnosis TV viewing		
	<1 hr/wk	1 to <3 hrs/wk	≥3 to <7 hrs/wk	≥7 hrs/wk	0 to 2 hrs/d	>2 to 4 hrs/d	>4 hrs/d
Number of participants	193	184	130	160	230	240	157
Age at exposure assessment, years, mean	72.3	70.6	71.0	71.2	70.9	71.6	71.8
Age at diagnosis, years, mean	68.9	67.0	67.1	67.4	67.2	68.1	67.9
Sex, %							
Men	66	68	77	82	69	79	69
Women	34	32	23	18	31	21	31
Non-Hispanic White, %	88.0	89.4	94.3	91.2	89.2	89.8	91.7
Smoking status, %							
Never smoker	32.2	33.0	33.6	29.9	35.7	29.56	26.2
Past smoker	46.7	46.7	58.1	53.1	49.5	52.4	52.1
Current smoker	7.0	6.5	0.7	4.3	2.7	5.9	8.5
College graduate/post graduate, %	28.4	36.4	50.8	47.9	44.6	40.4	28.7
Body mass index, kg/m^2^	29.44	28.53	28.11	27.70	28.2	27.7	30.2
Alcohol intake, g/d, mean	5.7	9.7	10.7	11.5	11.4	9.4	5.9
History of diabetes, %	15.6	10.0	8.9	9.9	7.6	11.8	15.3
History of high blood pressure, %	65	46.7	53.6	32.1	46.1	50.2	58.4
First course of treatment, %							
Surgery	36.8	43.2	51.0	40.9	47.0	38.2	39.9
Chemotherapy	2.1	3.4	2.8	0.6	1.3	3.3	1.7
Radiation	3.1	3.2	3.4	4.3	4.0	3.4	3.1
Immunotherapy	1.1	0.5	0	0.6	0.4	0.8	0
Tumor summary stage, %							
In situ	0	0	0	0.7	0.6	0	0
Localized	81.1	79.4	83.3	83.1	81.1	77.9	85.8
Regional metastases	14.7	18.7	16.7	14.0	16.7	18.8	12.8
Distant metastases	4.2	1.9	0	2.2	1.6	3.3	1.4

All values (except age) are standardized to the age distribution of the study population. Categories may not sum to 100% due to missing data. Covariates were assessed in the baseline questionnaire/risk factor questionnaire (hypertension variable), except smoking status and body mass index, which were assessed in the follow-up questionnaire.

### Moderate to vigorous physical activity

Increasing levels of moderate to vigorous physical activity showed a statistically significant inverse relation to all-cause mortality in the age- and sex-adjusted model and the multivariable-adjusted model [multivariable-adjusted HRs for <1 hr/wk (reference), 1 to <3 hrs/wk, ≥3 to <7 hrs/wk, and ≥7 hrs/wk = 1.0, 1.16 (95% CI = 0.78–1.72), 0.94 (95% CI = 0.60–1.49), and 0.60 (95% CI = 0.38–0.96; p-trend = 0.03), Table 2]. Additional control for BMI did not essentially change the results. The corresponding HRs for renal cell cancer mortality were 1.00 (reference), 0.96 (95% CI = 0.47–1.99), 1.07 (95% CI = 0.47–2.44), and 0.59 (95% CI = 0.25–1.38), p-trend = 0.33).

**Table 2:** Hazard ratios and 95% confidence intervals of mortality among individuals diagnosed with renal cell cancer according to post-diagnosis moderate to vigorous physical activity.

	Post-diagnosis physical activity				
	<1 hr/wk	1 to <3 hrs/wk	≥3 to <7 hrs/wk	≥7 hrs/wk	*p*-trend
**All-cause mortality**					
Deaths	56	55	33	31	
Model 1	1.00	1.07 (0.73–1.55)	0.85 (0.55–1.31)	0.62 (0.40–0.97)	0.02
Model 2	1.00	1.16 (0.78–1.72)	0.94 (0.60–1.49)	0.60 (0.38–0.96)	0.03
Model 3	1.00	1.15 (0.77–1.70)	0.95 (0.60–1.50)	0.60 (0.38–0.96)	0.03
**Renal cell cancer mortality**					
Deaths	21	16	10	10	
Model 1	1.00	0.83 (0.43–1.60)	0.72 (0.34–1.55)	0.57 (0.27–1.23)	0.15
Model 2	1.00	0.96 (0.47–1.99)	1.07 (0.47–2.44)	0.59 (0.25–1.38)	0.33
Model 3	1.00	0.92 (0.44–1.91)	1.04 (0.45–2.37)	0.57 (0.24–1.33)	0.30

Model 1: adjusted for age at exposure assessment (continuous), age at cancer diagnosis (continuous), and sex.

Model 2: adjusted for all variables in Model 1 and additionally adjusted for education (less than 12 yrs, 12 yrs, vocational training or some college education, college graduate/postgraduate, unknown), ethnicity (non-Hispanic White, non-Hispanic Black, other, unknown), history of diabetes (yes, no), history of hypertension (yes, no, missing), smoking from the follow-up questionnaire (never smoker, stopped smoking 10 or more years ago, stopped smoking 5–9 years ago, stopped smoking 1–4 years ago, stopped smoking within last year, currently smoking, unknown), alcohol consumption (0, 0.1 to 14.9, ≥15g/d), surgery (yes, no, unknown/missing), chemotherapy (yes, no, unknown/missing), radiation (yes, no, unknown/missing), stage (in situ, localized, regional metastases, distant metastases, unknown/not abstracted/missing), and total sitting time (0 to 5 hrs/d, 5 to 8 hrs/d, >8 hrs/d).

Model 3: adjusted for all variables in Model 2 and additionally adjusted for body mass index from the follow-up questionnaire (18.5-<25.0 kg/m^2^, 25.0-<30.0 kg/m^2^, 30.0-<35.kg/m^2^, 35-<65 kg/m^2^, missing).

In sensitivity analyses, the inverse association of physical activity and all-cause mortality was attenuated after excluding individuals who died within the first year of exposure assessment (multivariable-adjusted HR = 0.65, 95% CI = 0.39–1.07, p-trend = 0.047). Excluding participants diagnosed with renal cell cancer within one year of physical activity revealed a slightly stronger inverse association between physical activity and all-cause mortality (multivariable-adjusted HR = 0.56, 95% CI = 0.34–0.93, p-trend = 0.01).

### TV viewing and total sitting

TV viewing was positively associated with risk of all-cause mortality in the age- and sex-adjusted model, with HRs of 1.0, 1.25 (95% CI = 0.86–1.82), and 1.52 (95% CI = 1.02–2.26, p-trend = 0.04) for 0 to 2 hrs/d (reference), >2 to 4 hrs/d, and >4 hrs/d, respectively (Table 3). The risk estimate was attenuated and no longer statistically significant following additional control for other covariates (HR = 1.44, 95% CI = 0.92–2.24, comparing the extreme categories; p-trend = 0.11). Our results showed no indication for a relation of TV viewing time to renal cell cancer mortality (Table 3). Moreover, total sitting time showed no significant associations with mortality risk (Table 4).

**Table 3:** Hazard ratios and 95% confidence intervals of mortality among individuals diagnosed with renal cell cancer according to post-diagnosis TV viewing.

	Post-diagnosis TV viewing			*p*-trend
	0 to 2 hrs/d	>2 to 4 hrs/d	>4 hrs/d	
**All-cause mortality**				
Deaths	49	65	49	
Model 1	1.00	1.25 (0.86–1.82)	1.52 (1.02–2.26)	0.04
Model 2	1.00	1.24 (0.83–1.86)	1.44 (0.92–2.24)	0.11
Model 3	1.00	1.24 (0.83–1.86)	1.44 (0.92–2.25)	0.11
**Renal cell cancer mortality**				
Deaths	17	24	12	
Model 1	1.00	1.35 (0.72–2.53)	1.08 (0.51–2.26)	0.75
Model 2	1.00	1.42 (0.70–2.88)	1.02 (0.44–2.36)	0.90
Model 3	1.00	1.39 (0.68–2.84)	1.03 (0.45–2.40)	0.87

HR = hazard ratio, CI = confidence interval

Model 1: adjusted for age at exposure assessment (continuous), age at cancer diagnosis (continuous), and sex.

Model 2: adjusted for all variables in Model 1 and additionally adjusted for education (less than 12 yrs, 12 yrs, vocational training or some college education, college graduate/postgraduate, unknown), ethnicity (non-Hispanic White, non-Hispanic Black, other, unknown), history of diabetes (yes, no), history of hypertension (yes, no, missing), smoking from the follow-up questionnaire (never smoker, stopped smoking 10 or more years ago, stopped smoking 5–9 years ago, stopped smoking 1–4 years ago, stopped smoking within last year, currently smoking, unknown), alcohol consumption (0, 0.1 to 14.9, ≥15g/d), surgery (yes, no, unknown/missing), chemotherapy (yes, no, unknown/missing), radiation (yes, no, unknown/missing), stage (in situ, localized, regional metastases, distant metastases, unknown/not abstracted/missing), and moderate to vigorous physical activity (<1 hr/wk, 1 to <3 hrs/wk, ≥3 to <7 hrs/wk, and ≥7 hrs/wk).

Model 3: adjusted for all variables in Model 2 and additionally adjusted for body mass index from the follow-up questionnaire (18.5-<25.0 kg/m^2^, 25.0-<30.0 kg/m^2^, 30.0-<35.kg/m^2^, 35-<65 kg/m^2^, missing).

**Table 4:** Hazard ratios and 95% confidence intervals of mortality among individuals diagnosed with renal cell cancer according to post-diagnosis total sitting time.

	Post-diagnosis total sitting time			*p*-trend
	0 to 5 hrs/d	>5 to 8 hrs/d	>8 hrs/d	
**All-cause mortality**				
Deaths	43	50	71	
Model 1	1.00	1.05 (0.70–1.58)	1.16 (0.79–1.69)	0.45
Model 2	1.00	0.97 (0.63–1.50)	1.13 (0.74–1.71)	0.55
Model 3	1.00	1.00 (0.65–1.56)	1.16 (0.76–1.77)	0.48
**Renal cell cancer mortality**				
Deaths	15	20	19	
Model 1	1.00	1.24 (0.63–2.43)	0.88 (0.45–1.74)	0.73
Model 2	1.00	1.48 (0.70–3.15)	1.19 (0.54–2.62)	0.64
Model 3	1.00	1.51 (0.70–3.25)	1.24 (0.56–2.74)	0.57

HR = hazard ratio, CI = confidence interval

Model 1: adjusted for age at exposure assessment (continuous), age at cancer diagnosis (continuous), and sex.

Model 2: adjusted for all variables in Model 1 and additionally adjusted for education (less than 12 yrs, 12 yrs, vocational training or some college education, college graduate/postgraduate, unknown), ethnicity (non-Hispanic White, non-Hispanic Black, other, unknown), history of diabetes (yes, no), history of hypertension (yes, no, missing), smoking from the follow-up questionnaire (never smoker, stopped smoking 10 or more years ago, stopped smoking 5–9 years ago, stopped smoking 1–4 years ago, stopped smoking within last year, currently smoking, unknown), alcohol consumption (0, 0.1 to 14.9, ≥15g/d), surgery (yes, no, unknown/missing), chemotherapy (yes, no, unknown/missing), radiation (yes, no, unknown/missing), stage (in situ, localized, regional metastases, distant metastases, unknown/not abstracted/missing), and moderate to vigorous physical activity (<1 hr/wk, 1 to <3 hrs/wk, ≥3 to <7 hrs/wk, and ≥7 hrs/wk).

Model 3: adjusted for all variables in Model 2 and additionally adjusted for body mass index from the follow-up questionnaire (18.5-<25.0 kg/m^2^, 25.0-<30.0 kg/m^2^, 30.0-<35.kg/m^2^, 35-<65 kg/m^2^, missing).

In an evaluation of joint associations of physical activity and TV viewing, the combinations of regular physical activity and low levels of TV viewing or regular physical activity and high levels of TV viewing were related to reduced overall mortality risk among renal cell cancer survivors compared to low physical activity levels and high amounts of TV viewing (S1 Table). The same observation was made for total sitting time, although the inverse association was not statistically significant for the regular physical activity/low total sitting time group when compared with the low physical activity/high total sitting time viewing group.

We also analyzed change in physical activity or sedentary behavior from before to after diagnosis using pre-diagnosis exposure information from the risk factor questionnaire and post-diagnosis exposure information from the follow-up questionnaire. We found that participants who were physically active (≥4 hrs/wk) before and after diagnosis had a statistically significant reduced risk of all-cause mortality (multivariable-adjusted HR = 0.56, 95% CI = 0.32–0.96, S2 Table).

## Discussion

In this first investigation of physical activity in relation to risk of mortality among survivors of renal cell cancer, increasing levels of moderate to vigorous physical activity were associated with decreased risk of all-cause mortality, regardless of the level of sedentary behavior. In contrast, prolonged time spent sedentary was unrelated to risk of mortality.

Cancer survivors experience recurrence, second malignancies, and comorbid chronic conditions [28–30], including hypertension and diabetes mellitus, the latter of which are related to increased risk of renal cell cancer [31, 32]. Randomized controlled trials among survivors of obesity-related cancers have shown favorable insulin and insulin-like-growth factor (IGF), inflammation, and immune system responses to enhanced exercise [33–36]. Other putative biologic mechanisms linking physical activity to renal cell cancer survival involve improvements in blood pressure [6] and lipid peroxidation [7]. Thus, renal cell cancer survivors may be particularly sensitive to physical activity intervention in terms of survival.

A recent investigation in the NIH-AARP Diet and Health Study reported a positive trend for the association between physical activity and renal cell cancer mortality among healthy subjects at baseline [37]. One possible explanation is that the biologic mechanisms through which physical activity prior to diagnosis affects disease progression and how physical activity after diagnosis may affect recurrence are different. For example, while BMI has been found to increase risk of renal cell cancer incidence, better survival has been demonstrated in overweight patients after diagnosis of renal cell cancer [38], suggesting that risk factors and biologic mechanisms operating during the pre-diagnosis phase may not inevitably extrapolate to the post-diagnosis and treatment phase.

In the present study, TV viewing was associated with increased risk of all-cause mortality in the age- and sex-adjusted model, but that positive association was attenuated following additional control for other covariates. This finding suggests that the potential adverse health effects of TV viewing may be partly attributable to other factors related to prolonged TV viewing, such as high energy intake [39] and smoking behaviors [40, 41]. From a physiologic perspective, biologic pathways related to alterations in body fat mass, metabolic hormones, sex hormones, and chronic inflammation may mediate the relation of TV viewing to renal cell cancer survival [42].

Our study needs to be interpreted within the context of its strengths and limitations. Strengths of this study reside in its prospective design, long follow-up duration, and the availability of information on major potential confounding variables. Limitations of the present study include the self-report nature of our physical activity and sedentary behavior measures, which may have resulted in exposure misclassification. With almost one quarter of participants reporting at least 7 hrs/wk of moderate to vigorous physical activity, it is likely that physical activity after diagnosis was overestimated in our study population. There is also potential for confounding by unknown or poorly characterized variables. Surgery is the primary treatment for renal cell cancer. In our study, we controlled for cancer stage and the first course of treatment, however, we lacked information about secondary treatment. Participants who did not receive surgical treatment initially may experience cancer progression and receive secondary treatments affecting their physical activity behavior. Moreover, pre-existing illness may have resulted in unintended lower physical activity levels at baseline, raising concerns about reverse causality. In sensitivity analyses, the inverse relation of post-diagnosis physical activity to all-cause mortality was attenuated after excluding deaths within 12 months of the activity assessment. Finally, our analysis may have been hampered by marginally sufficient sample size, as evidenced by the wide CIs for some of the risk estimates.

In summary, our findings show that engaging in increased levels of moderate to vigorous physical activity is associated with lower risk of overall mortality among renal cell cancer survivors, suggesting that physical activity may improve survival of these patients. Current recommendations state that cancer survivors should comply with the physical activity guidelines for adults of at least 150 minutes per week of moderate physical activity [43]. Thus, cancer survivors should be counseled to avoid physical inactivity and to return to normal daily activities as early as possible depending on their medical and physical health conditions [44].

The role of sedentary behavior for cancer survival needs further clarification. Future research should incorporate objective activity assessments with multiple assessment time points into prospective cohort studies to improve physical activity and sedentary behavior measures and to determine the optimal dose and balance of physical activity and sedentary behavior for maximum health benefits [45, 46].

## Supporting information

S1 Fig Timeline of data collection and analytic follow-up. Renal cell cancer cases diagnosed between 1995/1996 and 2004/2005 were included. Follow-up started at the date of follow-up questionnaire entry and ended at the date of death or end of follow-up at December 31, 2011. Baseline questionnaire/risk factor questionnaire in 1995/1996: Assessment of covariates (except body mass index and smoking). Follow-up questionnaire in 2004/2005: Assessment of exposures and body mass index and smoking.
(PPTX)

S1 Table Joint associations of post-diagnosis physical activity, TV viewing, and total sitting time with all-cause mortality among survivors of renal cell cancer. (DOCX)

S2 Table Pre-to post-diagnosis changes in physical activity, TV viewing, and total sitting time with all-cause mortality among survivors of renal cell cancer. (DOCX)
