# Supplementary figures and images for: Physical activity and sedentary behavior in relation to mortality among renal cell cancer survivors
Source: PLoS One. 2018 Jun 12;13(6):e0198995. doi: 10.1371/journal.pone.0198995 (PMC5997343; doi:10.1371/journal.pone.0198995)

## Slide 1
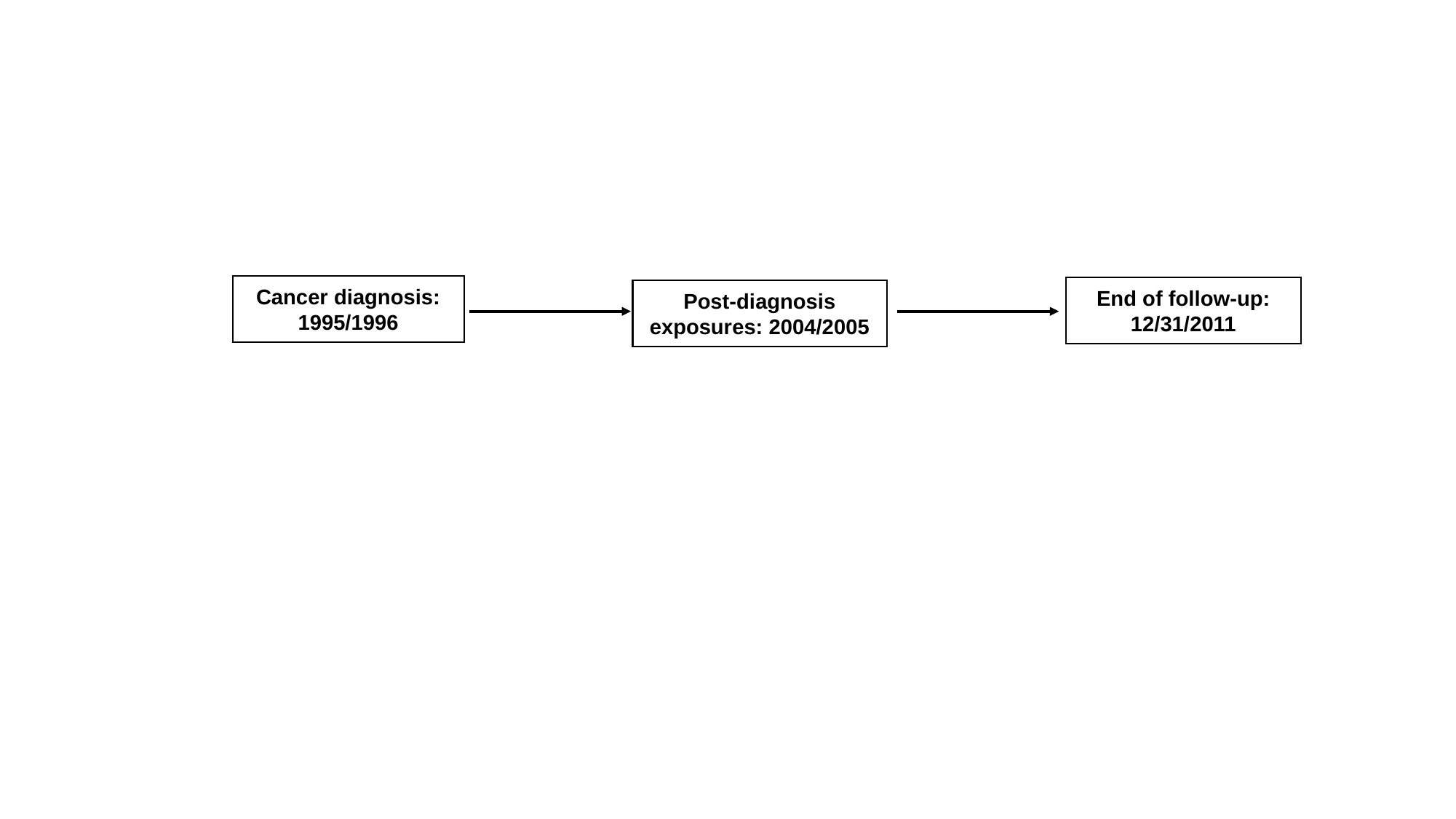

Cancer diagnosis: 1995/1996
End of follow-up:
12/31/2011
Post-diagnosis exposures: 2004/2005

Supplement: S1 Fig — Renal cell cancer cases diagnosed between 1995/1996 and 2004/2005 were included. Follow-up started at the date of follow-up questionnaire entry and ended at the date of death or end of follow-up at December 31, 2011. Baseline questionnaire/risk factor questionnaire in 1995/1996: Assessment of covariates (except body mass index and smoking). Follow-up questionnaire in 2004/2005: Assessment of exposures and body mass index and smoking. (PPTX) [file pone.0198995.s001.pptx]
