# Supplementary material for: Physical activity and sedentary behavior in relation to mortality among renal cell cancer survivors
Source: PLoS One. 2018 Jun 12;13(6):e0198995. doi: 10.1371/journal.pone.0198995 (PMC5997343; doi:10.1371/journal.pone.0198995)
Supplement: S1 Table — (DOCX) [file pone.0198995.s002.docx]

**S1 Table.** Joint associations of post-diagnosis physical activity, TV viewing, and total sitting time with all-cause mortality among survivors of renal cell cancer.

|  | | **TV viewing** |  | **Total sitting time** | |
| --- | --- | --- | --- | --- | --- |
| **Physical activity** |  | **High (>2 to hrs/d)** | **Low (≤2 hrs/d)** | **High (>5 to hrs/d)** | **Low (≤5 hrs/d)** |
| **Low (<4 hrs/wk)** | Deaths | 80 | 34 | 87 | 28 |
|  | Multivariable-adjusted HR (95% CI) | 1.00 | 0.78 (0.51-1.21) | 1.00 | 0.92 (0.59-1.45) |
| **High (≥4 hrs/wk)** | Deaths | 33 | 15 | 33 | 15 |
|  | Multivariable-adjusted HR (95% CI) | 0.59 (0.38-0.90) | 0.43 (0.23-0.78) | 0.56 (0.36-0.85) | 0.58 (0.32-1.05) |

HR=hazard ratio, CI=confidence interval, TV= television

Multivariable-adjusted models include age at exposure assessment (continuous), age at cancer diagnosis (continuous), sex, education (less than 12 yrs, 12 yrs, vocational training or some college education, college graduate/postgraduate, unknown), ethnicity (non-Hispanic White, non-Hispanic Black, other, unknown), history of diabetes (yes, no), history of hypertension (yes, no, missing), smoking from the follow-up questionnaire (never smoker, stopped smoking 10 or more years ago, stopped smoking 5-9 years ago, stopped smoking 1-4 years ago, stopped smoking within last year, currently smoking, unknown), alcohol consumption (0, 0.1 to 14.9, ≥15g/d), surgery (yes, no, unknown/missing), chemotherapy (yes, no, unknown/missing), radiation (yes, no, unknown/missing), and stage (in situ, localized, regional metastases, distant metastases, unknown/not abstracted/missing).
