# Supplementary material for: Physical activity and sedentary behavior in relation to mortality among renal cell cancer survivors
Source: PLoS One. 2018 Jun 12;13(6):e0198995. doi: 10.1371/journal.pone.0198995 (PMC5997343; doi:10.1371/journal.pone.0198995)
Supplement: S2 Table — (DOCX) [file pone.0198995.s003.docx]

**S2 Table.** Pre- to post-diagnosis changes in physical activity, TV viewing, and total sitting time with all-cause mortality among survivors of renal cell cancer.

|  | **Physical activity** | | | |
| --- | --- | --- | --- | --- |
|  | **Maintain low** | **Reduced** | **Increased** | **Maintain high** |
|  | (<4 hrs/wk) | (≥4 to <4 hrs/wk) | (<4 to ≥4 hrs/wk) | (≥4 hrs/wk) |
| Deaths | 49 | 31 | 9 | 22 |
| Multivariable-adjusted HR (95% CI) | 1.00 | 0.99 (0.60-1.64) | 0.50 (0.24-1.06) | 0.56 (0.32-0.96) |
|  | **TV viewing** | | | |
|  | (≥3 hrs/d) | (<3 to ≥3 hrs/d) | (≥3 to <3 hrs/d) | (<3 hrs/d) |
| Deaths | 20 | 16 | 11 | 58 |
| Multivariable-adjusted HR (95% CI) | 1.00 | 0.98 (0.47-2.04) | 1.14 (0.52-2.51) | 1.10 (0.62-1.94) |
|  | **Total sitting time** | | | |
|  | (<5 hrs/d) | (≥5 to <5 hrs/d) | (<5 to ≥5 hrs/d) | (≥5 hrs/d) |
| Deaths | 16 | 5 | 42 | 42 |
| Multivariable-adjusted HR (95% CI) | 1.00 | 0.87 (0.30-2.52) | 1.15 (0.59-2.24) | 1.33 (0.69- 2.55) |

HR=hazard ratio, CI=confidence interval, TV=television. Multivariable models are adjusted for age at exposure assessment (continuous), age at cancer diagnosis (continuous), sex, education (less than 12 yrs, 12 yrs, vocational training or some college education, college graduate/postgraduate, unknown), ethnicity (non-Hispanic White, non-Hispanic Black, other, unknown), history of diabetes (yes, no), history of hypertension (yes, no, missing), smoking (never smoker, stopped smoking 10 or more years ago, stopped smoking 5-9 years ago, stopped smoking 1-4 years ago, stopped smoking within last year, currently smoking, unknown), alcohol consumption (0, 0.1 to 14.9, ≥15g/d), surgery (yes, no, unknown/missing), chemotherapy (yes, no, unknown/missing), radiation (yes, no, unknown/missing), stage (in situ, localized, regional metastases, distant metastases, unknown/not abstracted/missing), and post-diagnosis moderate to vigorous physical activity (<1 hr/wk, 1 to <3 hrs/wk, ≥3 to <7 hrs/wk, and ≥7 hrs/wk) or total sitting time (0 to 5 hrs/d, 5 to 8 hrs/d, >8 hrs/d).
